# Effect of Curcumin Intake on Skeletal Muscle Oxygen Saturation Parameters in Older Participants

**DOI:** 10.3390/antiox13101175

**Published:** 2024-09-26

**Authors:** Olavo João Frederico Ramos-Junior, Vivian dos Santos Pinheiro, Tatiane Gomes dos Santos de Souza, Thiago Silveira Alvares

**Affiliations:** 1 Nutrition and Exercise Metabolism Research Group, Federal University of Rio de Janeiro, Macaé 27971-525, RJ, Brazil; olavite@gmail.com (O.J.F.R.-J.); vivianpinheiro14@gmail.com (V.d.S.P.); tatiane.6780@gmail.com (T.G.d.S.d.S.); 2 Food and Nutrition Institute, Multidisciplinary Center, Federal University of Rio de Janeiro, Macaé 27930-560, RJ, Brazil

**Keywords:** bioactive compounds, curcumin, muscle oxygen saturation, nitric oxide, aging

## Abstract

Introduction: Aging is associated with increased reactive oxygen species (ROS) and reduced bioavailability of nitric oxide (NO). Curcumin has been shown to increase NO bioavailability due to its ability to neutralize ROS, preventing oxidative stress. The present study aimed to investigate the effect of curcumin intake on skeletal muscle oxygen parameters and exercise tolerance in response to exercise in older people. Changes in circulating levels of NO metabolites were also investigated. Methods: Older subjects consumed 10 g of turmeric root extract from *Curcuma longa* L. (containing 95.33% of the total curcuminoids) or placebo in a randomized, double-blind, crossover study. A time of 2 h after ingestion, the participants performed one set of rhythmic handgrip exercise until the limit of tolerance, followed by 5 min of recovery. During exercise and exercise recovery, skeletal muscle oxygen saturation parameters were recorded. Results: During exercise, the amplitude of deoxyhemoglobin was greater after curcumin intake compared to placebo (CUR: 13.11 ± 9.52 vs. PLA: 10.22 ± 8.39 μM, *p* = 0.030). Furthermore, a faster skeletal muscle oxygen resaturation during exercise recovery was observed after curcumin compared to placebo (CUR: 1.01 ± 0.65 vs. PLA: 0.32 ± 0.20%.s^−1^, *p* = 0.004). These results were associated with significant changes in plasma nitrite (CUR: 6.82 ± 11.68 vs. PLA: −4.94 ± 17.28%, *p* = 0.028). There was no statistical difference in the total hemoglobin, exercise time until fatigue, and plasma nitrate between groups. Conclusions: The present study suggests that curcumin improves muscle oxygenation status at the capillary level in older adults by possibly improving muscle oxygen extraction and/or delivery, with no effect on exercise tolerance.

## 1. Introduction

Increased reactive oxygen species (ROS) in the vascular wall has been demonstrated with advancing age [1,2,3]. Major sources of ROS, including anion superoxide and other free radicals in blood vessels, are a membrane-associated NADH/NADPH oxidase expressed by endothelial and vascular smooth muscle cells and uncoupled endothelial nitric oxide synthase (eNOS) that can inactivate nitric oxide (NO) [3,4,5,6]. The bioactivity of anion superoxide has been shown to increase with aging [3,7]. For example, increased anion superoxide could reduce NO bioavailability and impair endothelial function with aging by reacting with NO to form peroxynitrite. In turn, peroxynitrite oxidizes tetrahydrobioterin to its inactive form, reducing NO synthesis and increasing anion superoxide production by eNOS [7,8]. These oxidative stress-induced biochemical events reduce NO bioavailability and impair NO biological functions.

Since NO may regulate blood flow [9], oxygen delivery to the skeletal muscle during and after exercise may be compromised in older people [10,11,12]. This may lead to fatigue, cramps, discomfort, or limb pain during daily activities, reducing the quality of life of older people. Therefore, investigation of nutritional strategies that improve NO bioavailability and skeletal muscle hemodynamic is of high clinical importance. 

Curcumin is a curcuminoid from *Curcuma longa* L. root and rhizome, commonly known as turmeric, and widely consumed as a spice ingredient, especially in Asian countries. Curcumin has gained popularity in the scientific community due to its antioxidant properties and positive effects on endothelial function [13,14,15,16,17]. The antioxidant property of curcumin is one of the main reasons for its cardiovascular benefits, due to its ability to neutralize ROS, preventing their interaction with NO and subsequent formation of oxidizing agents [18].

We recently found that acute supplementation with turmeric root extract (a natural source of curcumin) significantly increased cerebral oxygenation and blood volume during brain activation during dynamic exercise in older participants [19], suggesting that curcumin can contribute to improving cerebral hemodynamic in aging. Furthermore, curcumin has been demonstrated to induce mitochondrial biogenesis in skeletal muscle [20] and to improve the rate of oxygen consumption in dysfunctional isolated mitochondria [21], suggesting that curcumin intake may help to improve oxygen use during exercise. However, whether curcumin can increase skeletal muscle oxygen delivery and/or increase muscle capacity to extract or utilize oxygen in older adults has yet to be determined. Muscle oxygen saturation measured by near-infrared spectroscopy reflects the balance between muscle oxygen delivery and muscle oxygen extraction during exercise [22,23,24]. The recovery rate of muscle oxygen resaturation following the completion of exercise has been used as an index of deficient muscle oxygen delivery in relation to muscle oxygen demand [11].

The underlying mechanism for curcumin improving skeletal muscle oxygenation status at microvasculature is supposed to result from its antioxidant properties [15] since reduced NO bioavailability during aging may result from NO interaction with ROS. Therefore, maintaining redox balance against oxidant conditions through food nutrients with high antioxidant properties would improve NO bioavailability, which may positively affect the muscle oxygenation status and exercise tolerance in response to exercise.

Therefore, the present work aimed to evaluate whether a single dose of curcumin promotes changes in skeletal muscle oxygenation and blood volume at the microvascular level, as well as exercise tolerance, following the completion of a rhythmic exercise protocol. Changes in NO metabolites (plasma nitrate and nitrite) levels were also investigated. We hypothesized that circulating NO metabolites, muscle oxygenation, blood volume, and exercise tolerance would improve after curcumin intake in the older participants.

## 2. Methods

### 2.1. Participants

Six older male and four older female subjects (age: 67 ± 4 years; height: 1.65 ± 0.13 m; body weight: 80 ± 12 kg; body mass index: 30 ± 4 kg/m^2^) participated in the present study. Individuals were sedentary and had at least one of the following risk factors for cardiovascular disease: high blood pressure and/or high fasting glucose. Medications used by the participants included antihypertensive, hypoglycaemic agents, statins, and proton pump inhibitors. Participants were excluded if they were younger than 65 years old, smokers, showing any disease, such as liver, lung, and/or kidney failure, and presenting any musculoskeletal injuries in the upper limbs that make it impossible to perform physical exercise. All participants signed the consent form after being fully informed about the nature and purpose of the study. The study was approved by the ethics committee from the Federal University of Rio de Janeiro, Campus Macaé (CAAE: 97055018.1.0000.5699). Clinical trial registry: NCT04119752.

### 2.2. Experimental Design

**Figure 1:**
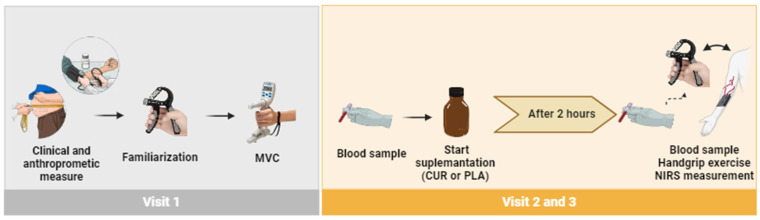
Experimental design of the study.

The study was randomized, crossover, placebo-controlled, and double-blind. All individuals came to the laboratory three times with 1-week intervals between visits. Participants were instructed to fast for at least 8 h, avoid nitrate-rich food, caffeine, vitamin and mineral supplements, alcohol intake, and physical exercise for at least 12 h before the testing visits. On the first visit, the participants’ clinical and anthropometric data were collected, and they were familiarized with the exercise protocol. In visits 2 and 3, after a 10-min period of quiet rest, the participants ingested 10 g of turmeric root extract from *Curcuma longa* L. (CUR) (containing 95.33% of the total curcuminoids) as a dietary source of curcumin, dissolved in 400 mL of water, or the same amount of corn starch as placebo (PLA). After 2 h of treatment intake, the exercise protocol was started, and NIRS parameters were evaluated (Figure 1). The beverages were offered in an amber bottle to blind the participant to know what condition (i.e., curcumin or placebo condition) they were in. A laboratory member not directly involved in the present study conducted and completed the randomization of the participants and blinding of the study. First, treatments A and B were coded as curcumin or placebo by tossing a coin. Then, curcumin and placebo drinks were portioned in amber bottles with similar appearance and consecutively numbered for each participant according to the randomization schedule. Each participant was assigned an order number and received the intervention in the corresponding bottle. Both the participants and the investigators were blinded to the treatment order. We chose to provide 10 g of turmeric root extract since such a dose is well tolerated when consumed orally and has been reported to improve cerebral oxygenation in older people [19].

The exercise consisted of rhythmic contractions of their forearm muscles at a controlled rate (120 contractions.min^−1^; 0.5 s:0.5 s contraction to relaxation duty cycle) and range of motion (10-cm excursion of the pulley wire with each contraction) using an adjustable hand grip strengthener. Each participant completed one 1-min exercise at 40% of the maximal voluntary contraction (MVC) until muscle fatigue. Participants were laid supine and used the dominant arm to perform the exercise. The MVC was taken during the first visit using a digital hand dynamometer (JAMAR, Model 5030J1, Sammons Preston Rolyan, Bolingbrook, IL, USA). Each MVC was 3 s in duration and separated by 30 s passive recovery to define the exercise workload. Exercise fatigue was determined when participants could not maintain the exercise rate (as contraction rhythm mismatch with metronome sound), and/or the range of motions was reduced for more than five consecutive contractions. Therefore, exercise time until fatigue (ETF) was recorded from the beginning of the exercise until fatigue and reported in seconds (s).

### 2.3. Muscle Oxygen Saturation Parameters

**Figure 2:**
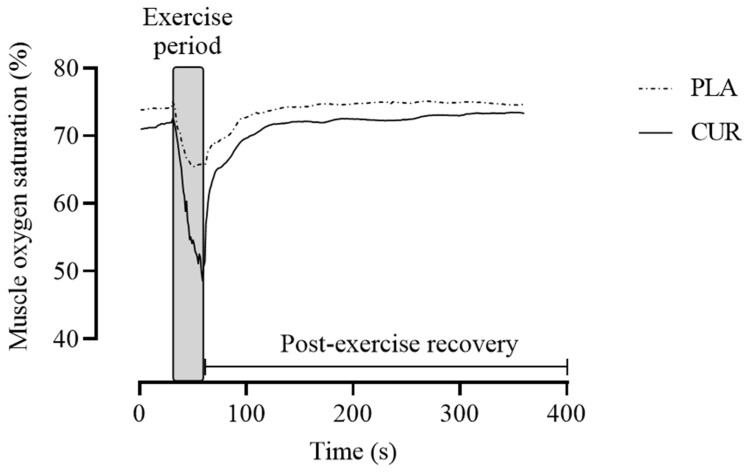
Representative profile of NIRS-derived measure of muscle oxygen saturation (SmO_2_) during the handgrip exercise protocol in both curcumin (CUR) and placebo (PLA) conditions.

Near-infrared spectroscopy (NIRS) was used as a non-invasive technique to assess relative changes in the concentrations of oxyhemoglobin ([O_2_Hb]) and deoxyhemoglobin ([HHb]), as well as the derived measures total hemoglobin ([tHb] = [O_2_Hb] + [HHb]) and muscle oxygen saturation (SmO_2_ = [O_2_Hb]/[tHb] × 100) within the skeletal muscle microvasculature [22,24,25]. The NIRS device (PortaMon, Artinis, Medical Systems, Elst, The Netherlands) was placed on each participant’s dominant forearm and surrounded by an elastic band to avoid external light interference in the NIRS signal. The NIRS system was connected to a computer via Bluetooth for data acquisition (10 Hz), and subsequent analysis of the raw data was performed using Oxysoft (version 3.0.103; Artinis Medical Systems, Elst, The Netherlands). The SmO_2_ and tHb recovery rates were calculated to assess the speed of muscle oxygen saturation and total hemoglobin recovery following exercise. The slope of a 5-s window of SmO_2_ and tHb immediately following the exercise’s end was used [25,26]. In addition, the amplitude of SmO_2_ and HHb during exercise was calculated to assess the magnitude of oxygen utilization during exercise. The amplitude of SmO_2_ during the recovery phase from exercise was calculated. A representative profile of NIRS-derived measures during all experimental protocol is shown in Figure 2.

### 2.4. Circulating NO Metabolites Analysis

The blood was drawn from antecubital veins and collected in EDTA-containing tubes, and then immediately centrifuged at 3000× *g* for 10 min at 4 °C to separate the plasma, before storing it at −80 °C for later analysis. NO production was assayed by measuring plasma nitrate and nitrite as previously described by Zhao et al. (2015) [27] by using a high-performance liquid chromatography (HPLC) system. In brief, 2 mL of plasma was mixed with 0.2 mL of 2 M sodium hydroxide and 0.4 g of zinc sulfate to remove proteins. The mixture was vortexed and then centrifuged at 4000× *g* for 10 min. The supernatant was collected, mixed with 250 mL of 0.17 M sulfanilic acid, and waited one minute. Afterward, 250 mL of 14 mM 1-naphthylamine was added to the mixture and diluted to the volume of 5 mL with deionized water. A volume of 0.4 mL of 10% Triton X-114 and 0.2 g ammonium sulfate were added to the sample, and it was placed in a water bath at 45 °C for 15 min. The samples were centrifuged at 4000× *g* for 10 min, and the surfactant-rich phase was collected and diluted with methanol. After filtering through a 0.45-mm nylon filter, 20 μL of sample was injected into the HPLC system. The HPLC system was equipped with a 5-μm reversed-phase C18 analytical column (L × I.D, 150 × 4.6 mm) guarded by a 5-μm reversed-phase C18 guard column (L × I.D, 50 × 4.6 mm) and a photodiode array detector model SPD-30MA (Shimadzu, Kyoto, Japan) monitoring the change in absorbance at 510 nm and 220 nm. The mobile phase had a flow rate of 1.0 mL/min and consisted of 15 mM sodium phosphate buffer (pH 7.5) and methanol.

### 2.5. Statistical Analysis

The Shapiro–Wilk test examined the data’s normality. A paired *t*-test was performed to identify differences in the investigated NIRS parameters (i.e., SmO_2_, tHb, and HHb) and exercise time until fatigue between CUR and PLA conditions. A paired *t*-test was also used to identify differences in percentage changes from pre- to post-intervention in plasma nitrate and nitrite between CUR and PLA groups. Cohen’s *d* was calculated as an effect size measurement, and the interpretation was based on the following benchmarks: *d* = 0.2, small effect; *d* = 0.5, medium effect; and *d* = 0.8, large effect. Statistical significance was assumed when *p* < 0.05. All analyses were performed using an available statistical package (IBM SPSS Statistics version 27 for Mac). Data were reported as mean ± standard deviation (SD).

## 3. Results

**Figure 3:**
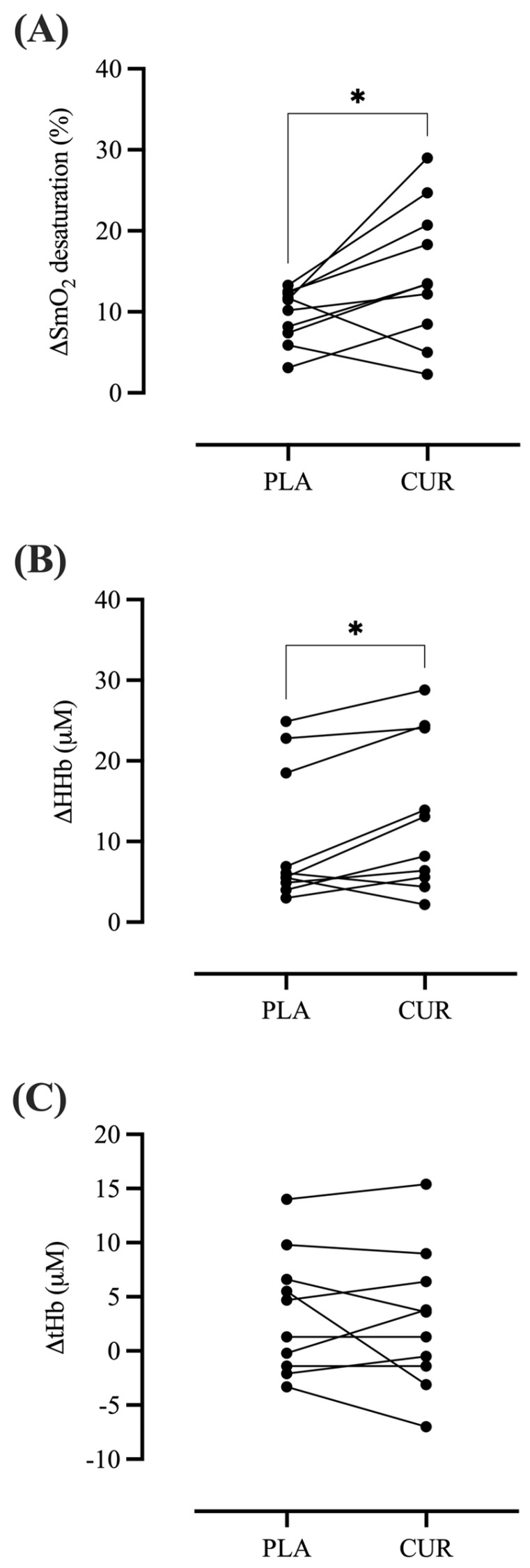
Changes in muscle oxygen desaturation (ΔSmO_2_) (**A**), amplitude of deoxyhemoglobin (ΔHHb) (**B**), and amplitude of total hemoglobin (ΔtHb) (**C**) during dynamic handgrip exercise in both curcumin (CUR) and placebo (PLA) conditions. The symbol * denotes significantly different from PLA (*p* < 0.05).

The curcumin dose was well-tolerated by all participants, with no reported adverse events or side effects. Table 1 shows the baseline characteristics of the participants enrolled in the present study. The individual values of the microvascular skeletal muscle oxygenation parameters evaluated during exercise and exercise recovery are shown in Figure 3. The levels of SmO_2_ (CUR: 73.02 ± 5.12 vs. PLA: 73.60 ± 5.53%, *p* > 0.05, *d* = 0.10), tHb (CUR: 4.50 ± 3.88 vs. PLA: 4.69 ± 3.78%, *p* > 0.05, *d* = 0.04), and HHb (CUR: −0.3 ± 2.2 vs. PLA: −0.7 ± 3.3%, *p* > 0.05, *d* = 0.63) were not statistically significant between CUR and PLA condition at rest (i.e., before beginning exercise). During exercise, the amplitude of muscle oxygen desaturation (ΔSmO_2_) (CUR: 14.76 ± 8.50 vs. PLA: 9.61 ± 3.34%, *p* = 0.043, *d* = 0.74) and deoxyhemoglobin (ΔHHb) (CUR: 13.11 ± 9.52 vs. PLA: 10.22 ± 8.39 μM, *p* = 0.030, *d* = 0.32) following CUR compared to PLA condition. There was no significant difference in total hemoglobin (ΔtHb) between groups (CUR: 2.75 ± 6.44 vs. PLA: 3.49 ± 5.63 μM, *p* = 0.529, *d* = 0.12).

**Figure 4:**
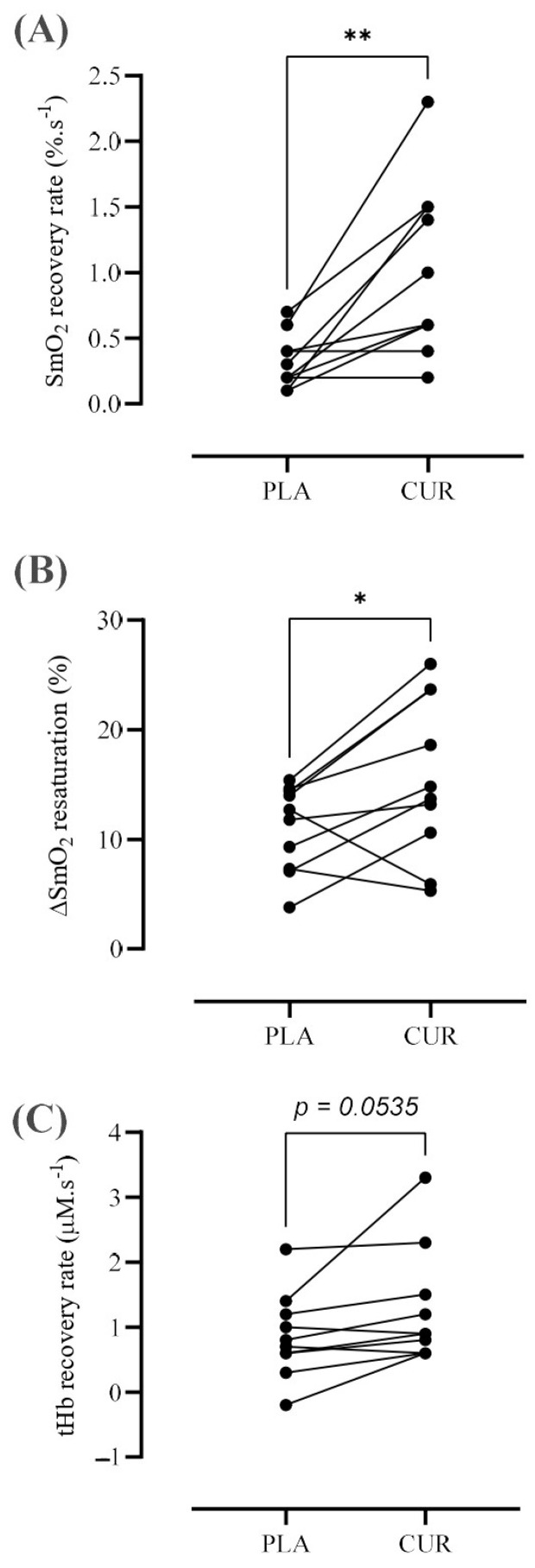
Changes in the muscle oxygen saturation (SmO_2_) recovery rate (**A**), the amplitude of muscle oxygen resaturation (ΔSmO_2_) (**B**), and total hemoglobin (tHb) recovery rate (**C**) after dynamic exercise in both curcumin (CUR) and placebo (PLA) conditions. The symbol * denotes significantly different from PLA (*p* < 0.05) and ** (*p* < 0.001).

The recovery rate of SmO_2_ (CUR: 1.01 ± 0.65 vs. PLA: 0.32 ± 0.20%.s^−1^, *p* = 0.004, *d* = 1.19) and the amplitude of SmO_2_ resaturation (CUR: 15.54 ± 7.32 vs. PLA: 11.04 ± 3.94%, *p* = 0.030, *d* = 0.70) were greater after curcumin intake as compared to the placebo condition (Figure 4). Also, tHb during exercise recovery tended to be faster in the CUR compared to PLA condition (CUR: 1.27 ± 0.89 vs. PLA: 0.86 ± 0.65%.s^−1^, *p* = 0.054, *d* = 0.51).

**Figure 5:**
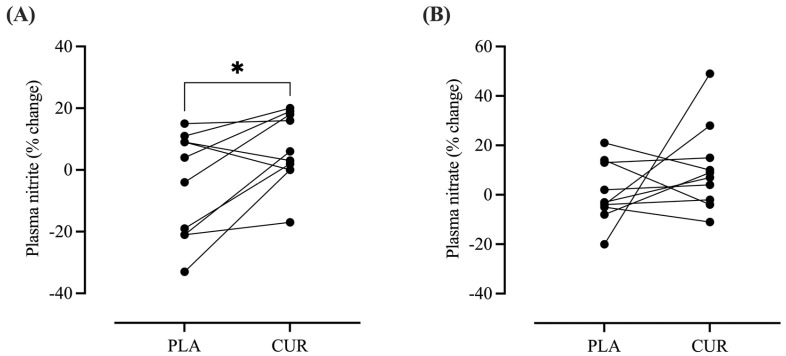
Percentage change from pre-supplementation values for plasma nitrite (**A**) and nitrate (**B**). The symbol * denotes significantly different from PLA (*p* < 0.05).

A significantly higher percentage change from pre-supplementation for plasma nitrite (∆nitrite) was found for CUR compared to PLA (CUR: 6.82 ± 11.68 vs. PLA: −4.94 ± 17.28%, *p* = 0.028, *d* = 0.77; Figure 5). There were no significant differences between CUR and PLA for plasma nitrate (CUR: 10.5 ± 17.11 vs. PLA: 0.71 ± 12.11%, *p* = 0.244, *d* = 0.64; Figure 5). Absolute values for plasma nitrate and nitrite are presented in Table 2.

There were no significant differences between CUR and PLA in ETF (CUR: 30.5 ± 6.60 s vs. PLA: 29.4 ± 6.83 s, *p* = 0.564, *d* = 0.16).

## 4. Discussion

This is the first investigation to examine the effects of a single dose of curcumin on forearm muscle oxygenation and blood volume at the microvascular level and exercise tolerance following a dynamic exercise protocol in older participants. The major finding of this study was that a single dose of curcumin improves muscle oxygen resaturation during exercise recovery in older participants. Furthermore, we observed greater muscle oxygen desaturation and oxygen extraction during the exercise protocol after curcumin intake compared to the placebo condition. These results were associated with increased plasma nitrite, suggesting improved NO bioavailability. 

An association between aging and reduced blood flow and oxygen delivery to the active muscle in older individuals has been documented [28,29,30,31]. The mechanisms underlying the attenuated hemodynamic response in aging can partly be explained by impaired NO availability [32]. Furthermore, NO plays a key role in mitochondrial biogenesis and may influence oxygen utilization through coupled cellular respiration functionally linked to enhanced ATP production [33]. Therefore, impaired NO availability in aging may compromise oxygen delivery/utilization by muscles during exercise. We recently reported that aging accompanied by risk factors for cardiovascular disease (i.e., high blood pressure, high fasting glucose, and dyslipidemias) shows a lower muscle oxygen resaturation rate than healthy young individuals [31]. Furthermore, Ichimura et al. (2006) [30] observed prolongation in the time of muscle reoxygenation following exercise in aging people, suggesting an impairment in muscle oxidative capacity and microvascular function in this population. Therefore, it should be expected that curcumin would improve NO availability and thus likely increase muscle perfusion and oxygen delivery to the muscle through its ability to neutralize ROS, preventing their interaction with NO to form peroxynitrites. In the present study, the muscle oxygen saturation rate following exercise was faster (*p* > 0.05) after curcumin intake compared to the control condition, which may indicate a greater balance between oxygen delivery relative to oxygen extraction by the muscle. In addition, total hemoglobin tended to be greater after curcumin intake than in the control condition but did not reach statistical significance (*p* = 0.058). The NIRS-derived total hemoglobin signal is related to blood volume changes in the microvasculature. It has been suggested to reflect changes in blood perfusion at the conduit artery level during exercise-induced hyperemia [25]. The mechanisms by which curcumin may improve skeletal muscle oxygenation are not fully understood. The most likely explanation came from studies reporting the ability of curcumin to induce changes in circulating NO metabolites (i.e., nitrate and nitrite) [15,34,35], suggesting an increase in NO bioavailability after curcumin supplementation. In the present study, we showed that curcumin improved NO synthesis as indicated by a significantly greater increment of ∆nitrite (% change from pre-supplementation) when participants were supplemented with curcumin compared to placebo. In agreement with our observations, previous studies have reported significant changes in circulating NO metabolites (i.e., nitrate and nitrite) after curcumin intake [15,34,35], suggesting that NO may have contributed to improving the muscle oxygenation status of the older participants. Although no significant changes were observed in plasma nitrate between curcumin and placebo conditions in the present study, it has been suggested that only plasma nitrite reflects changes in eNOS activity in the human body [36]. Despite evidence reporting that both plasma nitrate and nitrite can serve under certain conditions as quantitative metabolites of NO synthesis in humans, interpretation of the concentration of plasma nitrate and nitrite as a measure of potential NO synthesis needs to be performed carefully [37]. Since not only nitrite but also nitrate from food is a considerable contaminating contributor to changes in plasma nitrate and potentially to nitrite, the nitrate present in the diet may contribute to changes in plasma nitrate and nitrite without undergoing NO synthesis. This interference was mitigated in the present study by advising participants to avoid nitrate and nitrite-rich food the day before analysis. 

Aging has been associated with a marked impairment in skeletal muscle oxidative function during exercise [38,39,40]. For example, Chung et al. (2018) [40] demonstrated reduced forearm muscle oxidative function, measured by the recovery kinetics of muscle oxygen consumption using NIRS, in older compared to young participants, suggesting an overall reduction in mitochondrial function with age. In the present study, we observed a greater magnitude of deoxygenated hemoglobin response during the handgrip exercise after curcumin compared to placebo. Deoxygenated hemoglobin signal from NIRS during exercise has been used as an index of tissue oxygen extraction during exercise [38,39,40,41]. Our data corroborated with findings of a previous study [21] in which dietary supplementation with curcumin reduced mitochondrial dysfunction, increased ATPase activity, and restored oxygen consumption in isolated mitochondria of db/db mice, probably by scavenging free radicals and preventing protein and lipid oxidation. Also, other studies have already reported that controlling oxidative stress may be associated with improved mitochondrial function and oxygen delivery [20,42,43,44,45]. Panahi et al. (2012) [42] observed that curcumin supplementation for four weeks can increase the activity of superoxide dismutase, glutathione peroxidase, and catalase enzymes. According to the authors, the results may be partially explained by the effect of curcumin in neutralizing oxidative stress via multiple mechanisms, such as scavenging free radicals, increasing intracellular glutathione levels, inhibiting lipid peroxidation, and activating antioxidant enzymes. Although data from previous and present studies suggest that curcumin intake may improve muscle oxidative capacity during exercise in older individuals, more mechanistic studies in humans are needed to better elucidate this biological effect.

Besides the improvements in oxygen extraction and resaturation observed in the present study after curcumin intake, we found these improvements in muscle oxygenation status promoted a small effect size (i.e., *d* = 0.16) in the exercise tolerance of the older participants. However, compared to placebo, we observed an overall 4.5% increase in the exercise time until fatigue after curcumin intake. This may represent an important practical significance, considering that even minor improvements in muscle function could be worthwhile for older people, whose optimal muscular strength and endurance are essential in daily living and occupational activities.

## 5. Conclusions

The present study showed that curcumin improves forearm muscle oxygen extraction and resaturation at the microvascular level during exercise and recovery without affecting exercise tolerance. These results were associated with greater plasma nitrite response, which indicates that acute curcumin supplementation can positively affect skeletal muscle oxygenation status during exercise in older participants by potentially improving NO bioavailability.

## Figures and Tables

**Table 1:** Summary of baseline characteristics of the study participants.

Demographics	
n (male/female)	10 (6/4)
Age (years)	67 ± 4
Body mass (kg)	80 ± 12
Height (m)	1.65 ± 0.13
Body mass index (kg/m^2^)	30 ± 4
Clinical	
Total cholesterol (mg/dL)	163 ± 35
HDL-cholesterol (mg/dL)	40 ± 13
LDL-cholesterol (mg/dL)	104 ± 37
Triglycerides (mg/dL)	108 ± 37
Fasting glucose (mg/dL)	138 ± 57
Systolic blood pressure (mmHg)	131 ± 8
Diastolic blood pressure (mmHg)	81 ± 7
Heart rate (bpm)	66 ± 9

Values are presented as mean ± standard deviation. HDL, high-density lipoprotein; LDL, low-density lipoprotein.

**Table 2:** Values of plasma nitrate and nitrite concentrations before (Pre) and 2h after curcumin (CUR) or placebo (PLA) supplementation (Post).

Variable	PLA		CUR	
	Pre	Post	Pre	Post
Nitrate (µM)	44.25 ± 10.94	44.03 ± 10.16	43.09 ± 10.66	47.24 ±1 1.29
∆Nitrate (%)	0.71 ± 12.11		10.5 ± 17.11	
Nitrite (µM)	0.263 ± 0.233	0.264 ± 0.268	0.240 ± 0.255	0.257 ± 0.273
∆Nitrite (%)	−4.94 ± 17.28		6.82 ± 11.68 *	

Values are mean ± standard deviation. The symbol * denotes a statistically significant difference compared to PLA (*p* < 0.05).

## Data Availability

Data is contained within the article.
